# Demonstrating the value of medicines: evolution of value equation and stakeholder perception of uncertainties

**DOI:** 10.3402/jmahp.v4.31670

**Published:** 2016-07-20

**Authors:** Siva Narayanan

**Affiliations:** Market Access Solutions, LLC, Raritan, NJ, USA

**Keywords:** value, access, innovation, uncertainties

## Abstract

It is important to evaluate how the value of medicine is assessed, as it may have important implications for health technology and reimbursement assessments. The value equation could comprise ‘incremental benefit/outcome’ (relative results of care in terms of patient health, comparing the innovation to best available alternative(s)) in the numerator and ‘cost’ (relative costs involved in the full cycle of care (or a defined period) for the patient's medical condition, incorporating the relevant cost-offsets due to displacement of best available alternative(s)) in the denominator. This ‘relative value’ combined with the overall net budget impact (of including the drug in the formulary or reimbursed drug list) at the concerned population level in the given institution/region/country may better inform the usefulness of the new therapeutic option to the healthcare system. As product value messages are created, anticipating external stakeholder questions and information needs, including addressing three main categories of ‘uncertainties’, namely the scientific uncertainties, usage uncertainties, and financial uncertainties, could facilitate demonstration of optimal product value and help informed decision-making to benefit all stakeholders involved in the process.

Desire to improve efficiency in care delivery and achieve optimal outcomes with increasingly limited resources is in the minds of market access stakeholders globally (1–8). As regulatory agencies put forth stringent requirements for clinical evidence to approve products, these agencies have lately offered to open their doors for an early dialog with drug manufacturers related to design of clinical trials (including choice of subpopulation, endpoints, comparators, etc.) to help shape evidence generation for optimal patient outcomes and demonstration of relative (superior) value of the new intervention in relation to the alternatives (9). One of the ultimate goals is to eventually foster the development and expedite bringing new innovations into the market, to alleviate patient/societal burden in the concerned disease(s).

Marketing authorization of a product (the first step to market access) does not guarantee relevant approvals by payers or health governance organizations (HGOs) that are advised by health technology assessment bodies (HTABs); usefulness of the new therapeutic option to the healthcare system as assessed by payers/HGOs/HTABs (the second step to market access), thus, play a vital role in influencing use, adoption, and reimbursement of the new product in the market (10). Some countries have these two steps to market access efficiently cascaded in the product review/evaluation stage (e.g., Germany, Sweden), whereas a number of countries have them quite separate resulting in a lengthy process. In Europe, an EMA/HTA parallel scientific advice procedure does exist, and the pilot initiative has garnered accolades in introducing efficiency into the complex multi-stakeholder procedure involving multiple countries and agencies; however, it is recognized that there is more room for improvement (10, 11). In the United States, the aforementioned two steps of market access remain distinctly separate (and are expected to remain that way in the foreseeable future); following marketing authorization of a product, the private health plans, federally funded programs, pharmacy benefit managers, and hospitals have their own Pharmacy and Therapeutics committees to modulate access to medicines. Despite these differences in healthcare systems and delivery governance, resource constraints are shaping the healthcare decision-making around the globe at both macro and micro levels, necessitating rationing of resources to derive optimal ‘value’ (in terms of improved health outcomes and alleviated disease burden) from the healthcare investment.

It is universally recognized that a value-based approach will enable integration of multi-stakeholder perspectives in the evaluation of products and services, and their adoption (12–16). As Dr. Porter noted – ‘Value – neither an abstract ideal nor a code word for cost reduction – should define the framework for performance improvement in health care. Rigorous, disciplined measurement and improvement of value is the best way to drive system progress. Yet value in health care remains largely unmeasured and misunderstood’ (15). The value equation could comprise ‘outcomes’ (actual results of care in terms of patient health) in the numerator and ‘cost’ (total costs involved in the full cycle of care for the patient's medical condition) in the denominator (15). When payers/HGO/HTAB evaluate new products (innovation) entering the market, unless it is the first and only treatment option that will be available in the given therapy area, they are interested in the ‘relative value’ of the innovation more so than its absolute value (hence the payer/HGO/HTAB's increased focus on selection of relevant population and comparators in the clinical trials). In this scenario, the value equation could comprise ‘incremental benefit/outcome’ (relative results of care in terms of patient health, comparing the innovation to best available alternative(s)) in the numerator and ‘cost’ (relative costs involved in the full cycle of care (or a defined period) for the patient's medical condition, incorporating the relevant cost-offsets due to displacement of best available alternative(s)) in the denominator. This ‘relative value’ combined with the overall net budget impact (of including the drug in the formulary or reimbursed drug list) at the concerned population level in the given institution/region/country may better inform the second step to market access (usefulness of the new therapeutic option to the healthcare system). Some of the larger industrialized nations are starting to adopt these broad product assessments; the evaluations of the National Institute for Health and Clinical Excellence (NICE) of the United Kingdom (including the use of incremental cost-effectiveness ratio, which has its pros and cons) have some of these attributes (7, 14, 16, 17). Increased and standardized adoption of value evaluations across the countries is, however, needed both for HTA and rationing healthcare resources. As the value system evolves, one could also envision better incorporation of broader ‘societal benefit’ into the value equation in the future. As the value equation solidifies in the minds of stakeholders, value-based pricing and reimbursement concepts are likely to strengthen.

American Society of Clinical Oncology (ASCO) has recently made a credible attempt to introduce a revised value framework for cancer therapy assessment that has incorporated clinical benefit and toxicity, with or without additional consideration of improvements in disease symptoms and quality of life (QoL) and treatment-free intervals, based on disease status (advanced or adjuvant) (18). Drug acquisition cost and patient payment are also evaluated for a concerned regimen, to enable the patient to consider the net health benefit and the cost burden side-by-side. This value framework still lacks the incorporation of factors that patients may consider important, such as regimen convenience, impact on activities of daily living and QoL, and the ability to achieve personal and professional goals. This tool is likely to be useful to create personalized treatment plans for patients; however, projecting the health gains associated with a specific regimen/intervention at a population level and making decisions concerning health resource allocation at a macro level may prove difficult with this tool. Incorporation of societal benefit into this value framework, as it stands now, is impossible. Nevertheless, this experiment combined with other similar value assessment explorations such as the one by National Comprehensive Cancer Network (NCCN) in the United States which introduced Evidence Blocks™ (19) are small steps that may fuel the discussion of product value. This debate is bound to continue and evolve in therapy areas with high-priced drugs.

As product value messages are created, anticipating payer/HGO/HTAB questions and information needs could facilitate informed decision-making to benefit all stakeholders involved in the process. In essence, there are three categories of ‘uncertainties’ that one may need to focus on, namely scientific uncertainties, usage uncertainties, and financial uncertainties. The scientific uncertainties are associated with the facts that the risk–benefit profile of the innovation may change over the product life cycle, the clinical- and cost-effectiveness of the innovation may differ in real life (in comparison to what was demonstrated using clinical trial data), and the need for clarity concerning which patient will benefit the most, including who will respond to treatment in the real world. The usage uncertainties are associated with the facts that Healthcare Professionals (HCPs) may not always be able to target patient in which product is of (optimal) clinical value and that robust evidence communication may need to occur, and that there is potential for off-label use that modify the clinical and economic value proposition of the product. The financial uncertainties are associated with the questions – what is the optimal duration of treatment? (for the duration of an episode, or forever (in case of maintenance regimen)); which optimal product combinations may work and how it might impact the economic value proposition of the product?; and considering all of the above, what is the overall budget impact for year-1 versus future years, as the diagnostic and treatment dynamics evolve? If the aforementioned uncertainties are addressed effectively via robust evidence generation and communication, debate surrounding the price of medicines alone becomes a moot point, and the overall product value could be demonstrated effectively in the broader context.

The stakeholder needs continue to evolve, moving beyond the demonstration of product efficacy (does it work in clinical trials), and even beyond the demonstration of product effectiveness (does it work in the real world), to increasingly focus on its efficiency (does it contribute to more efficient use of resources). This is directly aligned with the creation of economic sustainability of healthcare system that is in the best interests of all stakeholders involved.
